# The genetic tumor background is an important determinant for heterogeneous *MYCN*‐amplified neuroblastoma

**DOI:** 10.1002/ijc.30050

**Published:** 2016-03-22

**Authors:** Dominik Bogen, Clemens Brunner, Diana Walder, Andrea Ziegler, Reza Abbasi, Ruth L. Ladenstein, Rosa Noguera, Tommy Martinsson, Gabriele Amann, Freimut H. Schilling, Marek Ussowicz, Martin Benesch, Peter F. Ambros, Inge M. Ambros

**Affiliations:** ^1^ Department of Tumor BiologyCCRI, Children's Cancer Research InstituteSt. Anna KinderkrebsforschungViennaAustria; ^2^ S^2^IRP, CCRI, Children's Cancer Research InstituteSt. Anna KinderkrebsforschungViennaAustria; ^3^ Department of PediatricsMedical University of ViennaViennaAustria; ^4^ Pathology DepartmentMedical School, University of Valencia, INCLIVAValenciaSpain; ^5^ Department of Clinical GeneticsInstitute of Biomedicine, Sahlgrenska Academy, University of GothenburgGothenburgSweden; ^6^ Department of Clinical PathologyMedical University of ViennaViennaAustria; ^7^ Pediatric OncologyOlgahospital, Klinikum StuttgartStuttgartGermany; ^8^ Department of Pediatric Oncology, Hematology and BMTWroclaw Medical UniversityWroclawPoland; ^9^ Division of Pediatric Hematology/OncologyDepartment of Pediatrics and Adolescent MedicineMedical University of GrazGrazAustria

**Keywords:** *MYCN* amplification, intratumoral heterogeneity, neuroblastoma, uniparental disomy

## Abstract

Amplification of *MYCN* is the signature genetic aberration of 20–25% of neuroblastoma and a stratifying marker associated with aggressive tumor behavior. The detection of heterogeneous *MYCN* amplification (hetMNA) poses a diagnostic dilemma due to the uncertainty of its relevance to tumor behavior. Here, we aimed to shed light on the genomic background which permits hetMNA in neuroblastoma and tied the occurrence to other stratifying markers and disease outcome. We performed SNP analysis using Affymetrix Cytoscan HD arrays on 63 samples including constitutional DNA, tumor, bone marrow and relapse samples of 26 patients with confirmed hetMNA by *MYCN*‐FISH. Tumors of patients ≤18m were mostly aneuploid with numeric chromosomal aberrations (NCAs), presented a prominent MNA subclone and carried none or a few segmental chromosomal aberrations (SCAs). In older patients, tumors were mostly di‐ or tetraploid, contained a lower number of MNA cells and displayed a multitude of SCAs including concomitant 11q deletions. These patients often suffered disease progression, tumor dissemination and relapse. Restricted to aneuploid tumors, we detected chromosomes with uniparental di‐ or trisomy (UPD/UPT) in almost every sample. UPD11 was exclusive to tumors of younger patients whereas older patients featured UPD14. In this study, the MNA subclone appears to be constraint by the tumor environment and thus less relevant for tumor behavior in aggressive tumors with a high genomic instability and many segmental aberrations. A more benign tumor background and lower tumor stage may favor an outgrowth of the MNA clone but tumors generally responded better to treatment.

## Abstract 2

**What's new?**

*MYCN* amplification (MNA) in neuroblastoma (NB) generally associates with an aggressive tumor behavior and detection of MNA leads to an automatic upstaging of the tumor in non‐stage 1 tumors. But what if only a fraction of the tumor cells is *MYCN*‐amplified? To investigate the diagnostic importance of heterogeneous MNA, the authors conducted a genetic analysis of samples from 26 NB patients with a particular focus on accompanying genetic aberrations. They concluded that tumor behavior is largely dependent on patient age and other chromosomal alterations in the genetic tumor background rather than the mere presence of the MNA clone.

**Abbreviations**
BM: bone marrow
BWS: Beckwith‐Wiedemann syndrome
CR: complete remission
CTX: chemotherapy
DOD: death of disease
DOS: death of surgery
DTC: disseminated tumor cell
dx: diagnosis
FISH: fluorescence *in situ* hybridization
hetMNA: heterogeneous *MYCN* amplification
homMNA: homogeneous *MYCN* amplification
INSS: International Neuroblastoma Staging System
MACS: magnetic activated cell sorting
MNA: *MYCN* amplification
NB: neuroblastoma
NCAs: numeric chromosomal aberrations
no mat: no material available
PR: partial response
SCAs: segmental chromosomal aberrations
tcc: tumor cell content
UDP: uniparental disomy
UTP: uniparental trisomy
wc: whole chromosome

## Introduction

Amplification of the oncogenic transcription factor *MYCN* is a stratifying marker in neuroblastoma (NB),^1^ the most common extracranial solid tumor in childhood. *MYCN* amplification (MNA) defines one of the most aggressive tumor subgroups comprised of 20–25% of all primary tumors^2^ and is associated with advanced stage, metastatic behavior, accelerated tumor progression and an overall poor prognosis.^3^, ^4^, ^5^, ^6^ MNA occurs more often in patients older than 12 months of age^7^, ^8^ and even among highly aggressive stage 4 tumors, *MYCN* status adds to distinguish subgroups with a worse prognosis.^9^ Near‐diploidy and the presence of only a few segmental chromosomal aberrations (SCAs), most prominently but not exclusively, a loss of the distal p‐arm of chromosome 1 and a gain of 17q, associate frequently with MNA in NB.^10^, ^11^, ^12^, ^13^, ^14^ With only few accompanying mutations and chromosomal alterations, MNA is considered an early, tumor‐driving event. Deletions at 11q occur rarely in tumors with homogeneous MNA (homMNA) and mark another unfavorable subgroup of NB.^15^, ^16^, ^17^

Determination of the prognostic relevance of a genetic marker in NB is generally based on the premise that an aberration is present in most if not all tumor cells. Until the advent of fluorescent *in situ* hybridization (FISH), Southern blot analysis was conducted to detect MNA in NB which suggested a high intratumoral penetrance and consistency of MNA in samples taken simultaneously or consecutively from primary and metastatic sites from the same patient.^18^ Detecting MNA at single‐cell resolution by FISH eventually overhauled this assumption and led to the identification of tumors exhibiting intratumoral heterogeneous *MYCN* amplification (hetMNA) in either scattered tumor cells or focal areas^19^, ^20^ but also with regard to time and location.^21^, ^22^, ^23^, ^24^ As only a few studies on hetMNA NBs have been published with ambiguous results,^23^ clear implications on the relevance of hetMNA to the clinical behavior of a tumor have yet to be determined. Patients presenting with hetMNA often receive the same risk stratification as homMNA patients and are allocated to the same high‐risk treatment protocol. The lack of biological and clinical understanding of hetMNA tumors may thus result in overtreatment and unnecessary exposure of ≤18m patients in particular to severe long‐term side effects.

Recently, Berbegall *et al*. began to shed light on the genetic composition of hetMNA tumors.^25^ In this study, we aimed to further illuminate the genomic background of hetMNA NB by SNP array analysis as well as investigate the association with other clinically relevant factors and markers.

## Material and Methods

### Patient samples

Patient samples were collected at the Children's Cancer Research Institute between 1994 and 2015. The heterogeneous *MYCN* status was confirmed by FISH analyses in all cases (for confirmatory FISH‐images of MNA cells see Supporting Information Figs. 1a and 1b). NB tumors were staged according to the International Neuroblastoma Staging System (INSS).^26^, ^27^ Ethical permission for the diagnostic analysis was granted by local ethics commissions. Bone marrow (BM) samples on microscope slides were stained for GD2 to detect NB cells and were subsequently analyzed for *MYCN* copy number by *MYCN*‐FISH after relocating GD2‐positve cells using an automatic fluorescence microscope.^28^ For group comparisons, data of 242 additional NB tumors from the Children's Cancer Research Institute's database with available SNP array data were included in this study. This cohort was an unbiased, consecutive collection of patient tumor samples without a selection for sex and age of the patient or tumor stage and location and was conform to the general distribution of these three clinical parameters.

### FISH

*MYCN*‐interphase FISH experiments for the detection of MNA heterogeneity were performed on either tumor touch preparations, sections or BM smears as described by Ambros *et al*.^20^ *MYCN* amplification was defined as a 4‐fold increase of the *MYCN* signal number compared to the reference probe located on chromosome 2 in accordance with Ambros *et al*.^29^ MNA heterogeneity was defined as the coexistence of these *MYCN*‐amplified as well as non‐amplified tumor cells in the same tumor.^29^ Internal standards were either the centromere‐specific D2Z (Oncor, Gaithersburg) or 2p probe (kind gift from M. Rocchi, University of Bari, Bari, Italy). DAPI was used to counterstain nuclei.

### DNA extraction and SNP array analysis

DNA extraction and SNP array analysis were performed as described by Ambros *et al*.^30^ In short, DNA was extracted from all samples including fresh or fresh‐frozen tumor‐containing samples as well as formalin‐fixed and paraffin embedded sections and BM cytospins applying the “high salt” extraction method.^24^, ^31^ Cells were resuspended in nuclei lysis buffer (10 mM Tris‐HCl, 400 mM NaCl and 2 mM EDTA, pH 8.2) and digested at 56°C overnight with a solution containing 20% SDS and proteinase K (20 mg/ml). DNA was then precipitated with 6M NaCl and microcentrifugation followed by the addition of 100% ethanol and microcentrifugation. For some of the BM samples with <50% tumor cell infiltrate, the tumor cell fraction was enriched by using an anti‐GD2 antibody and magnetic activated cell sorting (MACS, Miltenyi Biotec) prior to DNA extraction.^24^ DNA of BM samples marked with “cyto+” was extracted from BM cytospins after soaking the cells in PBS and scraping with a pipette tip. The Cytoscan HD (Affymetrix, UK) array was used for all samples and data analysis performed with the ChAS software package (Affymetrix, UK).

### Statistical analysis

Depending on the sample size, two‐sided Fisher's exact or Chi‐squared testing was performed using GraphPad Prism 5.0 to examine the association of the *MYCN* status and occurrence of uniparental di‐/trisomies within the hetMNA NB tumors as well as patient tumors in the database, respectively.

## Results

### Clinical characteristics of hetMNA patient cohort

**Figure 1:**
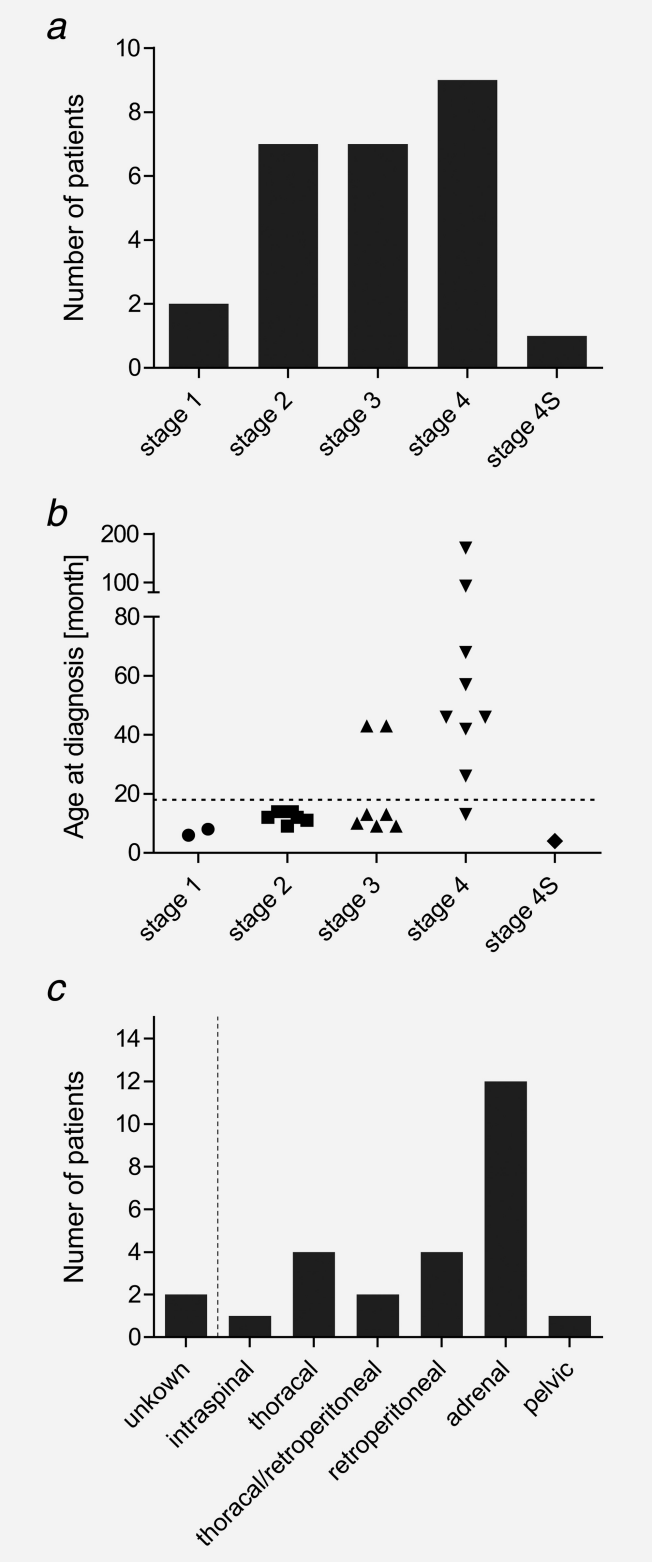
Clinical data associated with hetMNA in sample cohort. (*a*) Assigned stages of hetMNA tumors at diagnosis. (*b*) Age at diagnosis of the patients associated with clinical stage of the hetMNA tumors. The dashed line marks the 18‐month cutoff. Stages 2A and 2B were combined to stage 2 in subfigures *a* and *b*. (*c*) Localizations of primary tumors at diagnosis. The dashed line separates NB tumors with unknown location from those with a known primary location.

Patients in the hetMNA cohort were predominantly younger than the prognostically significant 18‐months cutoff (16/26, 62% versus 10/26, 38%) (Table 1). We denoted a slightly higher proportion of female (16/26, 62%) over male patients (10/26, 38%). Patient age at diagnosis ranged from 4 to 171 months with a median age of 14 months. Based on the INSS, tumor stages ranged from low to high with nine patients assigned to lower stages 1 and 2 and a predominance of higher stages 3 (7/26) and 4 (9/26) (Fig. 1 *a*). One tumor was diagnosed as stage 4S. All patients with stage 1, 2, or 4S tumors were younger than 18 months. On the contrary, the majority of stage 4 tumors (8/9, 89%) were diagnosed in patients older than 18 months (Fig. 1 *b*). Excluding two tumors with unknown primary localization, most primary tumors were found in the adrenal gland (12 out of 24). Other locations included retroperitoneal (4), thoracal (4), thoracal/retroperitoneal (2), intraspinal (1) and pelvic regions (1) (Fig. 1 *c*). Relapses and disease progression occurred in six patients and was confined to the >18m subgroup. Pat. #22 and #23 relapsed twice. Patient #26 showed no signs of remission before his death, thus we categorized him as “in progression”. Among these seven patients with disease progression, MNA was only detected heterogeneously in a liquor sample at the time of the first relapse of patient #22 (5 MNA cells vs. 28 non‐MNA cells in 27,000 analyzed cells). Three of these patients presented with bone marrow infiltration of non‐MNA tumor cells. The lung metastasis (323‐TU a and b) of patient #18 was also negative for MNA cells. The MNA status of the respective bone marrow and metastasis could not be assessed for the two remaining patients as material was not available.

**Table 1:** Clinical data of hetMNA neuroblastoma patients

Pat. #	Sex	Age at dx [months]	Stage	Localization of primary	BM infiltration at dx	MNA in BM	CTX	Disease progression	Outcome
1	F	4	4s	Adrenal	No	No	Yes	No	CR
2*	F	6	1	Unknown	No	No	No	No	CR
3	F	8	1	Adrenal	No	No	No	No	CR
4*	F	9	2A	Pelvic	No	No	Yes	No	CR
5*	F	9	3B	Unknown	No mat	No mat	–	–	DOS
6	F	9	3 (unresectable)	Adrenal	No	No	Yes	No	CR
7	F	10	3	Adrenal	No	No	Yes	No	CR
8	M	11	2B/4s/4	Thoracal	No	No	Yes	No	CR
9	M	12	2A	Thoracal/retroperitoneal	Yes	No	Yes	No	CR
10	M	12	2A	Adrenal	No	No	Yes	No	CR
11	F	13	4	Adrenal	Yes	No	Yes	No	PR
12	F	13	2/3	Retroperitoneal	No	No	Yes	No	CR
13	F	13	3	Retroperitoneal	Yes	No	Yes	No	CR
14	F	14	2	Thoracal	No	No	No	No	CR
15	F	14	2A	Thoracal	No mat	No mat	Yes	No	CR
16	F	14	2A	Thoracal	No	No	Yes	No	CR
17	M	26	4	Adrenal	Yes	Yes	Yes	No	Under therapy
18	F	42	4	Adrenal	No	No	Yes	Metastatic	Progression
19	M	43	3	Retroperitoneal	No	No	Yes	Local	DOD
20	F	43	3	Adrenal	No	No	Yes	No	CR
21	M	46	4	Adrenal	Yes	No mat	Yes	No	CR
22	M	46	4	Adrenal	Yes	Yes	Yes	Metastatic (2 relapses)	under relapse therapy
23	M	57	4	Intraspinal	Yes	No	Yes	Metastatic (2 relapses)	DOD
24	F	68	4	Retroperitoneal	Yes	No	Yes	Metastatic	DOD
25	M	93	4	Thoracal/retroperitoneal	Yes	No	Yes	Progression	DOD
26	M	171	4	Adrenal	Yes	No	Yes	No remission achieved	DOD

*Patients discovered during the Austrian NB screening program from 1991–97.^42^ Abbreviations: dx, diagnosis; BM: bone marrow; MNA: *MYCN* amplification; CTX: chemotherapy; No mat, no material available; CR, complete remission; PR, partial response; DOD, death of disease; DOS, death of surgery.

At the time of publication, two patients were still undergoing treatment and six had succumbed to the disease or surgical complications. However, the majority of hetMNA NB patients (17/26, 65%) especially in the younger group (15/16, 94%) responded well to treatment. Three of the younger patients fared well even without chemotherapy. Remission was achieved partially in one and completely in 16 patients.

### Spatial and temporal *MYCN* heterogeneity and the association with tumor cell ploidy

**Figure 2:**
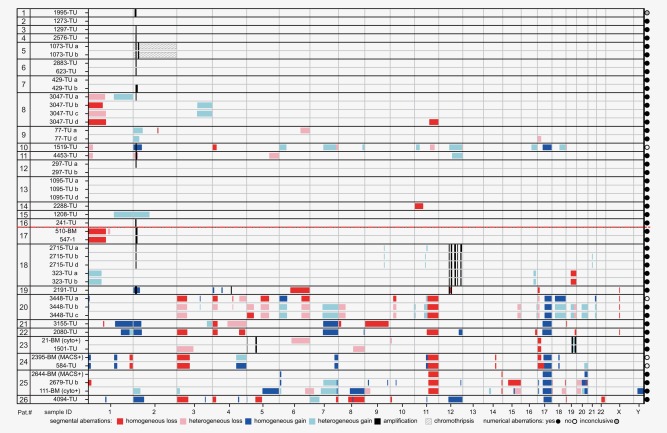
Summary of segmental chromosomal aberrations (>3MB) detected by SNP array analysis in hetMNA neuroblastoma samples. Samples are sorted according to patient age at diagnosis. The red dashed line marks the 18‐month age cutoff. Horizontal lines depict larger chromosomal aberrations and vertical lines amplifications or smaller deletions. Coloring of the boxes as described in the figure legend is based on the copy number state of the segments as determined by visual inspection of the SNP array data with the ChAS software. Presence of numerical aberrations in each sample is indicated by the fill of the circles to the right. Sequential breaks which result in the same type of aberration as classified in the legend but show different changes in copy number are indiscernible in the illustration. Due to the limited space, amplicons in close proximity cannot be visualized as separate entities (e.g., Pat.# 18, chromosome 12). For a detailed list of breaks and aberrations see *Supporting Information Table 2*. BM, bone marrow sample; cyto+, DNA was extracted from BM cytospin; MACS+, DNA from GD2‐positive cell fraction isolated by MACS from BM samples; TU, tumor sample.

The MNA clone detected by *MYCN*‐FISH was found by SNP array analysis in only 14 out of 26 patients (Fig. 2). Eleven patients belonged to the younger age group, who generally presented with tumors with a higher fraction of MNA cells. The percentage of MNA tumor cells in the remaining 12 samples was under the SNP array detection limit. We found intratumoral MNA heterogeneity as well as intertumoral heterogeneity in respect to location and time. The MNA clone in patient #23, for instance, was detectable by FISH in all GD2‐positive cells in the BM but not in the tumor sample after therapy. SNP analysis of either sample showed no sign of MNA (SNP array data of the BM samples are not displayed due to a low tumor cell content (tcc) of the sample and a flat profile). On the contrary, only two out of nine GD2‐ positive disseminated tumor cell (DTC) samples showed MNA. In patient #18, MNA was found in all three primary tumor pieces (2715‐TU) but not in the biopsy of the lung metastasis (323‐TU) collected six months later. Intratumoral hetMNA was detected by FISH and a small *MYCN* peak by SNP analysis in different pieces of a single sample of three patients (#7, #8 and #12) in the ≤18m cohort. Furthermore, composition and size of the *MYCN* amplicons varied profoundly between patients. The minimum region of overlap between the samples only contained *MYCN*, *MYCNOS* and *SNORA40* (Supporting Information Fig. 2).

DNA content was measured by flow cytometry or by the chromosomal copy numbers indicated by the SNP data in consideration of the tcc. Five tumors were diploid and one tetraploid whereas the remaining samples were aneuploid (Table 2, Supporting Information Table 1). All six patients with di‐ and tetraploid tumors were older than 18 months whereas the majority of patients with aneuploid tumors (15/19) were younger (Table 2).

**Table 2:** Ploidy and age distribution according to *MYCN* status in the NB patient cohort

*MYCN* status			Ploidy			Age		
	Count	Freq. [%]		Count	Freq. [%]		Count	Freq. [%]
			di‐/tetra	74	38.9	≤ 18 months	13	17.6
						> 18 months	55	74.3
						na	6	8.1
						Subtotal	74	100
			Aneuploid	95	50	≤ 18 months	49	51.6
Non‐MNA	190	70.9				> 18 months	37	38.9
						na	9	9.5
						Subtotal	95	100
			na	21	11.1	≤ 18 months	5	23.8
						> 18 months	13	61.9
						na	3	14.3
						Subtotal	21	100
			Subtotal	190	100			
			Di‐/tetra	5	19.2	≤ 18 months	0	0
						> 18 months	5	100
						na	0	0
hetMNA	26	9.7				Subtotal	5	100
			Aneuploid	21	80.8	≤ 18 months	16	76.2
						> 18 months	5	23.8
						na	0	0
						Subtotal	21	100
			na	0	0			
			Subtotal	26	100			
			Di‐/tetra	34	65.4	≤ 18 months	14	41.2
						> 18 months	20	58.8
						na	0	0
						Subtotal	34	100
			Aneuploid	14	26.9	≤ 18 months	3	21.4
homMNA	52	19.4				> 18 months	10	71.4
						na	1	7.1
						Subtotal	14	99.9
			na	4	7.7	≤ 18 months	0	0
						> 18 months	3	75
						na	1	25
						Subtotal	4	100
			Subtotal	52	100			

### Heterogeneous *MYCN*‐amplification is accompanied by a plethora of other segmental chromosomal aberrations in older but not in young patients

**Figure 3:**
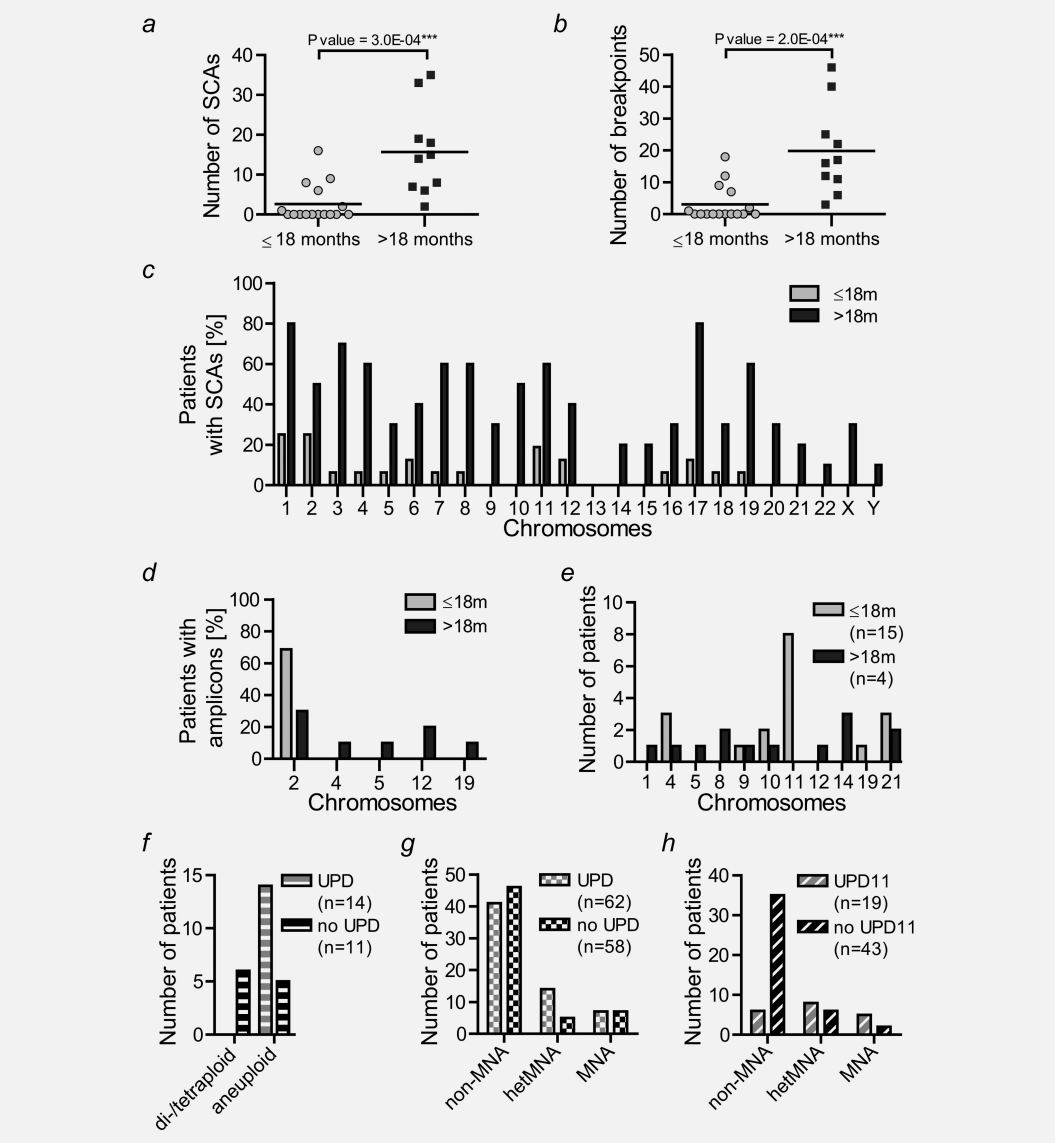
SCAs, amplicons and UPDs. Number of segmental chromosomal aberrations (*a*) and breakpoints (*b*) in 26 hetMNA tumors was significantly different between the two patient age groups. Percentages of patients with indicated chromosomes affected by segmental aberrations (*c*) and gene amplifications (*d*). Incidences of SCAs in any tumor cell‐containing sample were considered once per patient. (*e*) The stacked column chart shows the distribution of uniparental whole chromosome isomies in the patient cohort. Presence of UPDs or UPTs were counted as an event regardless of penetrance in the tumor. Patients were grouped based on age at diagnosis: ≤18 months and >18 months. (*f*) UPD occurrence in 25 hetMNA tumors with UPD data grouped by tumor ploidy: di‐/tetraploid (6) and aneuploid (19). (*g*) UPD occurrence in 120 aneuploid NB tumors in the CCRI database grouped by *MYCN* status: non‐MNA (87), hetMNA (19) and MNA (14). (*h*) occurrence of UPD11 among the tumors of the 62 patients that carried UPDs: non‐MNA (41), hetMNA (14) and MNA (7). Numbers in round brackets represent patient counts.

To investigate hetMNA‐associated chromosomal aberrations, we analyzed a total of 48 tumor‐cell‐containing samples (Fig. 2, Supporting Information Table 1). To exclude constitutional aberrations, we included 15 tumor‐free BM or peripheral blood samples as references for cases with aberrations smaller than 3 MB. In total, we detected 199 distinct SCAs (not including additional amplicons) affecting all chromosomes except for chromosome 13 (Fig. 2). Tumors of older patients displayed a significantly higher number of SCAs (mean of 15.7, range 2–35) and chromosomal breakpoints (mean of 19.8, range 3–46) than those of younger patients (SCAs: mean of 2.6, range 0–16; chromosomal breakpoints: mean of 3.1, range 0 to 18) (Figs. 3 *a* and 2 *b*). SCAs were found to be heterogeneous, occurring in only a fraction of tumor cells in samples of 13 patients. Most affected by SCAs were chromosomes 1 (4/15, 27%) and 2 (4/15, 27%) in young and chromosomes 1 (8/10, 80%) and 17 (8/10, 80%) in older patients (Fig. 3 *c*). Deletions at the 11q‐arm were found in tumors of five patients and never associated with a SNP array‐detectable MNA signal in the same sample. In addition to MNA, we identified other amplicons on chromosomes 2, 4, 5, 12, and 19, mostly in the heavily rearranged tumors of older patients (Fig. 3 *d*). Coamplifications of MDM2 and CDK4 on chromosome 12 were found in samples of two patients (Supporting Information Table 2). Signs of chromothripsis were found for chromosome 2 in the tumor of patient #5 (Supporting Information Fig. 3). Homozygous *ATRX* deletions were present in three out of the ten >18m patients but not in tumors of the young patients (Supporting Information Fig. 4). In comparison, none of the 52 homMNA but 17 out of 97 non‐MNA NB patients in our database displayed an intragenic deletion of *ATRX* (data not shown).

Due to the limited sensitivity of the SNP arrays and the intratumoral differences in clonal composition of the samples, we cannot unambiguously exclude the presence of additional heterogeneous SCAs and amplifications in subclones of very low frequencies.

### Whole chromosome uniparental di‐/trisomies associate with hetMNA in patients with aneuploid tumors

In our patient cohort, we identified a total of 31 somatic, whole chromosome uniparental disomies (wc‐UPDs) or –trisomies (wc‐UPTs) in 14 of 25 patients (∼73% of patients in young cohort, 30% in older cohort) (Fig. 3 *e*, Supporting Information Fig. 5). In three cases, UPDs were found to be heterogeneous (#8, #10 and #17). The tcc was too low to draw any UPD‐related conclusions in the one sample of patient #1. UPDs/UTPs were only found in aneuploid tumors (14/19) and often affected more than one chromosome per tumor (9/15) (Fig. 3 *f*). None of the six patients with di‐ or tetraploid tumors presented uniparental whole chromosomes (Fig. 3 *f*). This prevalence was also observable in the non‐MNA (158) and homMNA (45) NB tumors with available ploidy data in our database. Of the 102 patients with di‐/tetraploid tumors (71 non‐MNA and 31 homMNA tumors), only one non‐MNA tumor featured a wc‐UPD18 with unusual amplicons and gains (data not shown). On the contrary, UPDs were found in 48% (48/101) of aneuploid non‐MNA and homMNA tumors (Supporting Information Table 3). Although the association between *MYCN* status and occurrence of UPD within the aneuploid cohort was not significant [χ^2^ (2, *N* = 120) = 4.422, *p* = 0,1096], aneuploid hetMNA tumors had a higher UPD frequency with 74% (14/19) compared to 47% (41/87) of non‐MNA and 50% (7/14) of homMNA NB (Fig. 3 *g*). We also discovered SCAs affecting UPD‐chromosomes in hetMNA tumors of four patients (Supporting Information Fig. 6).

Detected in 40% (8/19) of all patients with aneuploid hetMNA tumors, UPD11 was not only the predominant uniparental disomy in this group but also associated exclusively with age ≤18 months (8/15, 53%). On the contrary, UPD of chromosome 14 was most prominent in aneuploid tumors of older patients (3/4, 75%). In aneuploid NBs with UPDs, we detected a significant association between *MYCN* copy number and occurrence of UPD11 [χ^2^ (2, *N* = 62) =15.05, *p* = 0,0005]. Whereas UPD11 was detected in 57% of heterogeneous (8/14) and 71% of homogeneous (5/7) aneuploid MNA tumors with UPDs, only 15% (6/41) of non‐MNA NBs featured this aberration (Fig. 3 *h*). Of note, none of the hetMNA NB patients with UPD11 relapsed and patients either responded partially to treatment (1/8) or are in complete remission (7/8).

## Discussion

Since the first discovery,^19^, ^20^, ^32^ intratumoral heterogeneity of MNA in NB has been investigated more extensively by only a few research groups.^23^, ^25^ This study provides an unbiased examination of the genomic background of hetMNA NB in 26 patients. We found aneuploidy in hetMNA tumors of 19 patients including 15 ≤18m patients. This association of young age and aneuploidy is in accordance with the literature.^33^, ^34^ Likewise, NCA‐only profiles, which are an attribute of favorable tumors with a low risk and an overall good prognosis,^9^ were confined to tumors of this age group. These patients responded well to therapy (two patients did not recieve cytotoxic therapy) and achieved partial responses or complete remission without relapses despite featuring a prominent MNA clone. In contrast, all six patients with di‐/tetraploid tumors belonged to the older cohort. In contrast to MNA heterogeneity in this study, homMNA is more often associated with di‐/tetraploidy which is in accordance with the predominance of di‐/tetraploid homMNA tumors (65%) in our database (Table 2).^8^, ^33^, ^34^, ^35^, ^36^ HetMNA tumors of older patients were often highly aberrated with a multitude of SCAs but only contained a very small percentage of MNA cells. In accordance with previously published data,^25^ we detected a frequent association of hetMNA and 11q‐deletion.

Uniparental di‐ or trisomies (also referred to as copy‐neutral LOH) arise when one parental copy of a chromosome is lost while the other is subsequently reduplicated. An alternative mechanism is the loss of the non‐duplicated chromosome in a trisomy. Aside from cancer‐prone developmental diseases such as Beckwith‐Wiedemann syndrome (BWS) and Angelman syndrome, UPDs in children can be found in a variety of cancers including leukemias, rhabdomyosarcoma, retinoblastoma, nephroblastoma and NB (reviewed in Refs. 37 and 38). In a recent report, 35% of 134 examined primary NBs showed signs of UPD including wc‐UPDs in 35% of the cases.^39^ Occurrence of UPD was tightly associated with aneuploidy in our patient cohort (52%, 62/120) and disproportionally high in hetMNA tumors (74%, 14/19). Chromosome 11 was most frequently affected by wc‐UPDs/UPTs in 8 out of 14 patients with UPDs and exclusive to the younger age group. UPD11 in the context of NB may be of particular interest as the loss of heterozygosity of the q‐arm of chromosome 11 is a signature SCA of a highly aggressive subgroup of tumors and associates with older patient age and poor prognosis.^16^, ^40^ However, there appears to be a fundamental difference between segmental LOH resulting in a partial monosomy and a copy‐neutral wc‐UPD which likely concerns the balance in copy number of affected genes. In contrast to 11q‐deleted tumors, patients with UPD11‐containing hetMNA NB tumors responded well to treatment and had beneficial outcomes. However, the high survival rate may as well be tied to a low tumor stage and the young age of these patients as the outcome of the few homMNA NB patients with UPD11, who were all older than 18 months, was poor (data not shown). Without obtaining additional data on gene expression, mutations and the methylation pattern of chromosome 11, which was not the scope of this paper, we were unable to pinpoint a gene that could be potentially involved in NB oncogenesis. Yet, the occurrence of wc‐UPDs in general and UPD11 in particular warrants further investigation. To our knowledge, a similarly high frequency of a particular wc‐UPD has thus far not been reported in a subgroup of a cancer. wc‐UPDs could be of prognostic value in NB as was shown for serous ovarian cancer.^41^

**Figure 4:**
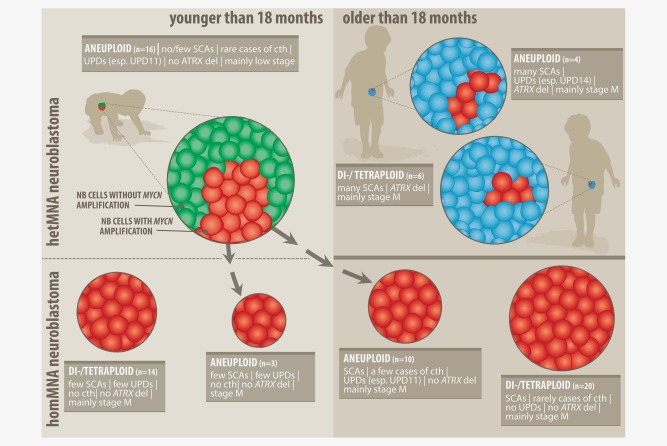
Hypothetical model of proposed tumor evolution of hetMNA tumors. This model is based on the comparison of clinical and biological parameters of hetMNA NB tumors (25) included in this study and homMNA tumors (47) with evaluable SNP array data collected at the CCRI. Green cells depict favorable *MYCN* non‐amplified tumor cells in tumors of ≤18m patients whereas blue cells represent non‐MNA NB cells with a highly rearranged genome and a multitude of SCAs in tumors of older patients. Red illustrates MNA cells. Arrows depict a possible transition of aneuploid hetMNA tumors into aneuploid homMNA tumors which can be suspected based on similarities in the genetic background. The fact that most aneuploid homMNA tumors were found in >18m patients would suggest a time‐dependent clonal takeover of homMNA cells.

To conclude, multilocational sampling, which may eventually include the implementation of liquid biopsy techniques, and a meticulous tumor work‐up are necessary to fully characterize NB genetically and to discover intratumoral heterogeneity of potentially risk‐stratifying markers. Technological advances in genomic profiling of single cells as well as bulk tumors will aid in improving the understanding of the complexity of NB. In addition our data corroborate the necessity to include single‐cell techniques in the diagnosis of NB and the detection of MNA or SCAs. The MNA subclone, for instance, was only detectable by FISH but missed by SNP array analysis in numerous cases due to the detection limit of the arrays. Furthermore, our results indicate that the behavior of hetMNA tumors more strongly depends on the genetic composition of the tumor background than the mere presence of the MNA subclone. In a tumor with largely favorable characteristics, the MNA clone appears to be less aggressive or is possibly lacking full malignancy resulting in a good therapeutic outcome of the patients. However, aneuploid MNA cells may eventually outgrow other subclones without adequate treatment. We found aneuploid homMNA tumors in our database which shared clinical and biological features with aneuploid hetMNA tumors of ≤18m patients (Fig. 4). Patients with these homMNA tumors were mostly older than 18 months which would support the notion of a potential clonal takeover with time. The contribution of the MNA clone to the overall tumor behavior in an aggressive genomic environment may be limited and the competition with other subclones may stifle its outgrowth.In line with this assumption, homMNA tumors in our registry rarely occur with 11q deletion and almost never present with a comparable number of aberrations or intragenic ATRX deletions contrary to aggressive hetMNA tumors in >18m patients (Fig. 4). Both the genetics of the ancestral clone and the timing of the occurrence of MNA during tumor evolution determine the relevance of an MNA subclone in NB. Larger studies with more patients are needed to corroborate our findings and fully solve the diagnostic challenges posed by intratumoral heterogeneity of MNA in NB, particularly in patients younger than 18 months of age.

## Supporting information

Supporting Information

Supporting Information Table 2.
