# Supplementary material for: The genetic tumor background is an important determinant for heterogeneous MYCN‐amplified neuroblastoma
Source: Int J Cancer. 2016 Mar 22;139(1):153–63. doi: 10.1002/ijc.30050 (PMC4949549; doi:10.1002/ijc.30050)
Supplement: Supplementary file 1 — Supporting Information [file IJC-139-153-s001.docx]

# Supporting Information

### Supporting Information Figure 1


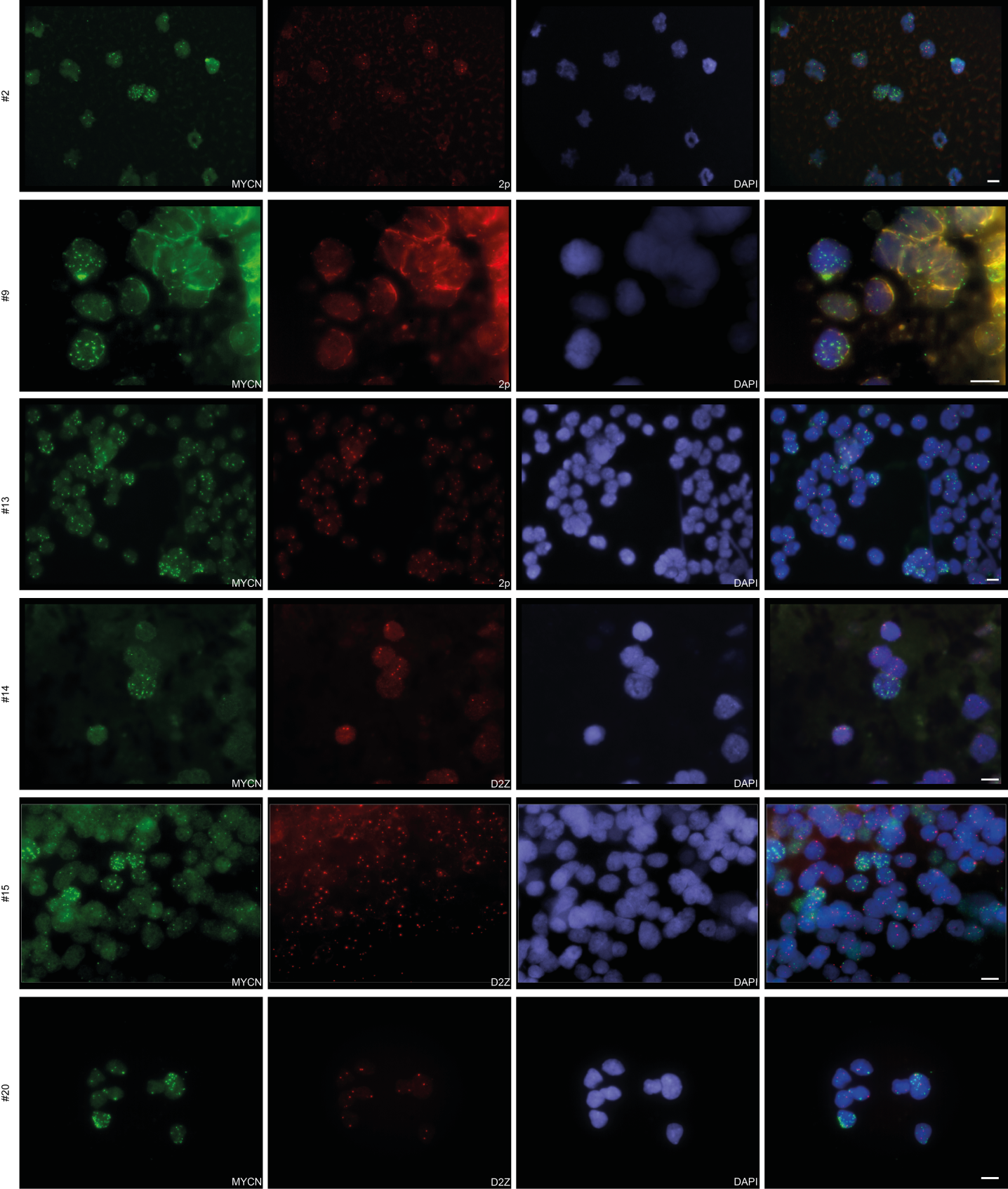


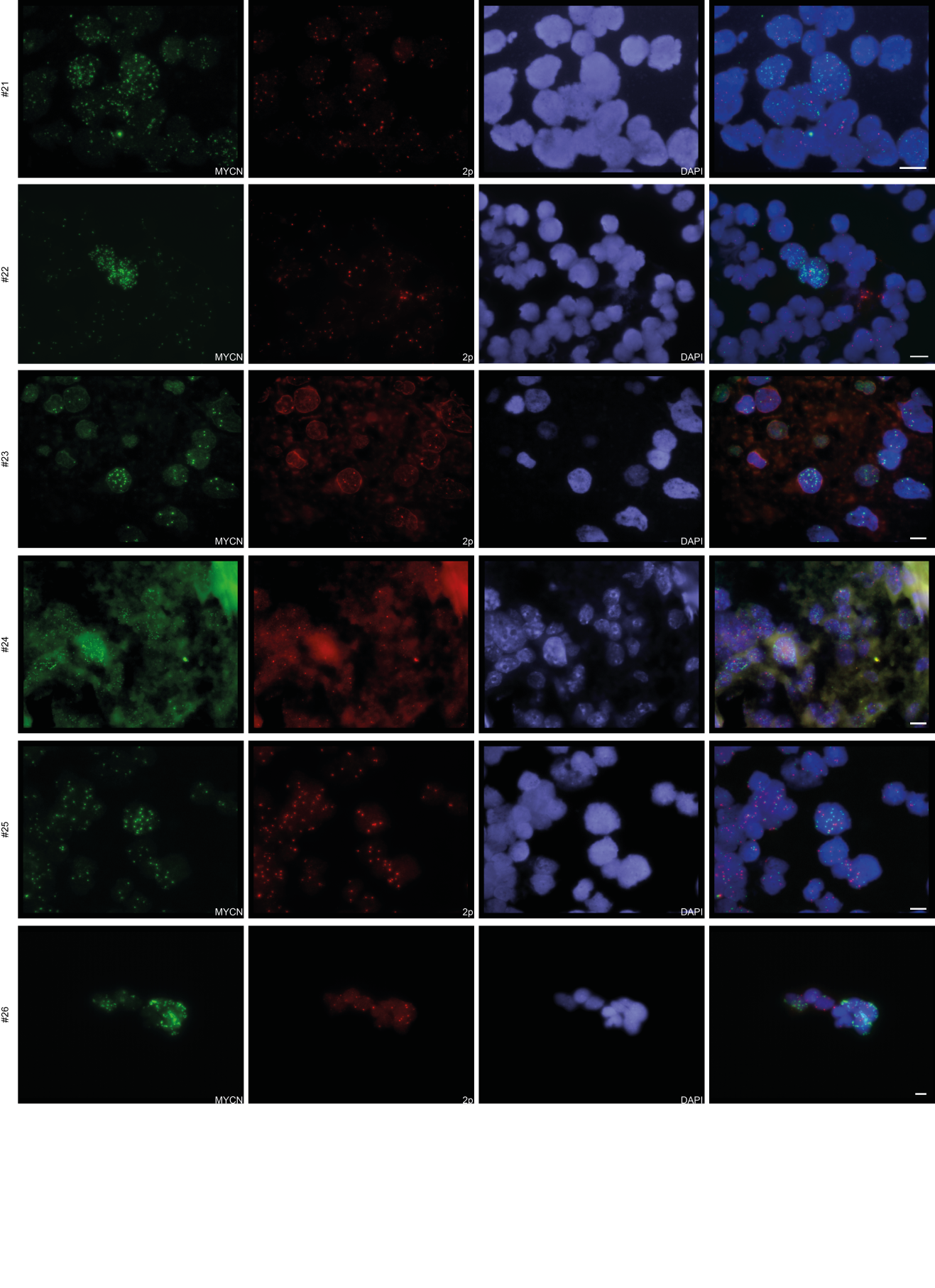


Confirmation of MNA cells on cryosections, paraffin sections or tumor touch preparations by *MYCN* interphase FISH (green channel) for patients without detectable MNA in bulk tumor DNA by SNP array analysis. Probes against 2p or D2Z were used as references (red channel) as indicated for each patient. DAPI was used to counterstain the nucleus (blue channel). The rightmost column shows merged images of the three channels. As images were taken at different magnifications the length of the bar can vary but is always 10µm.

### Supporting Information Figure 2


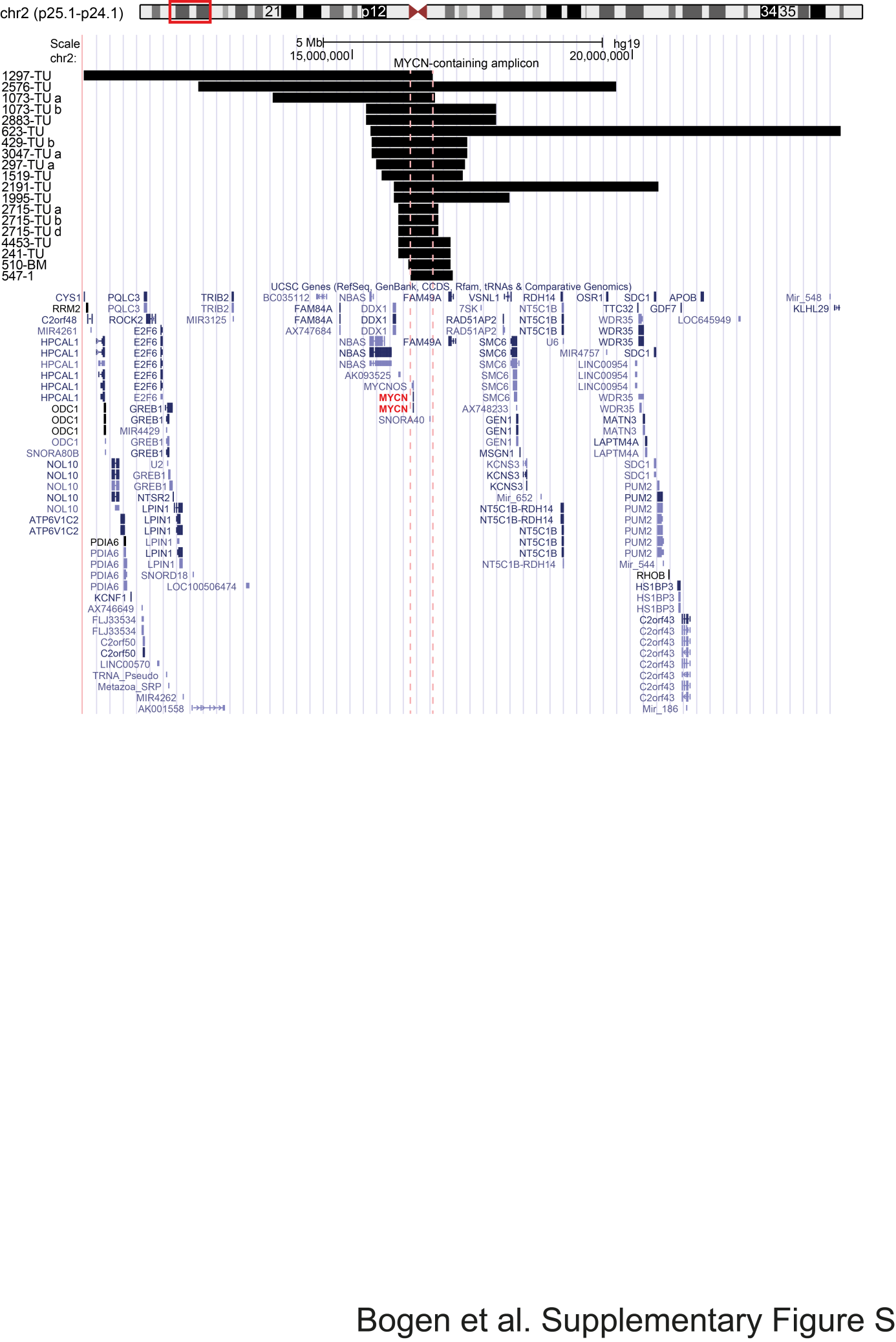


*MYCN*-amplicons as detected by SNP genotyping mapped by the UCSC genome browser (hg19). The minimum region of overlap of the amplicon between the samples is marked by pink dashed lines and only included *MYCN* accompanied by non-coding RNA genes *MYCNOS* and *SNORA40*.

###

###

### Supporting Information Figure 3


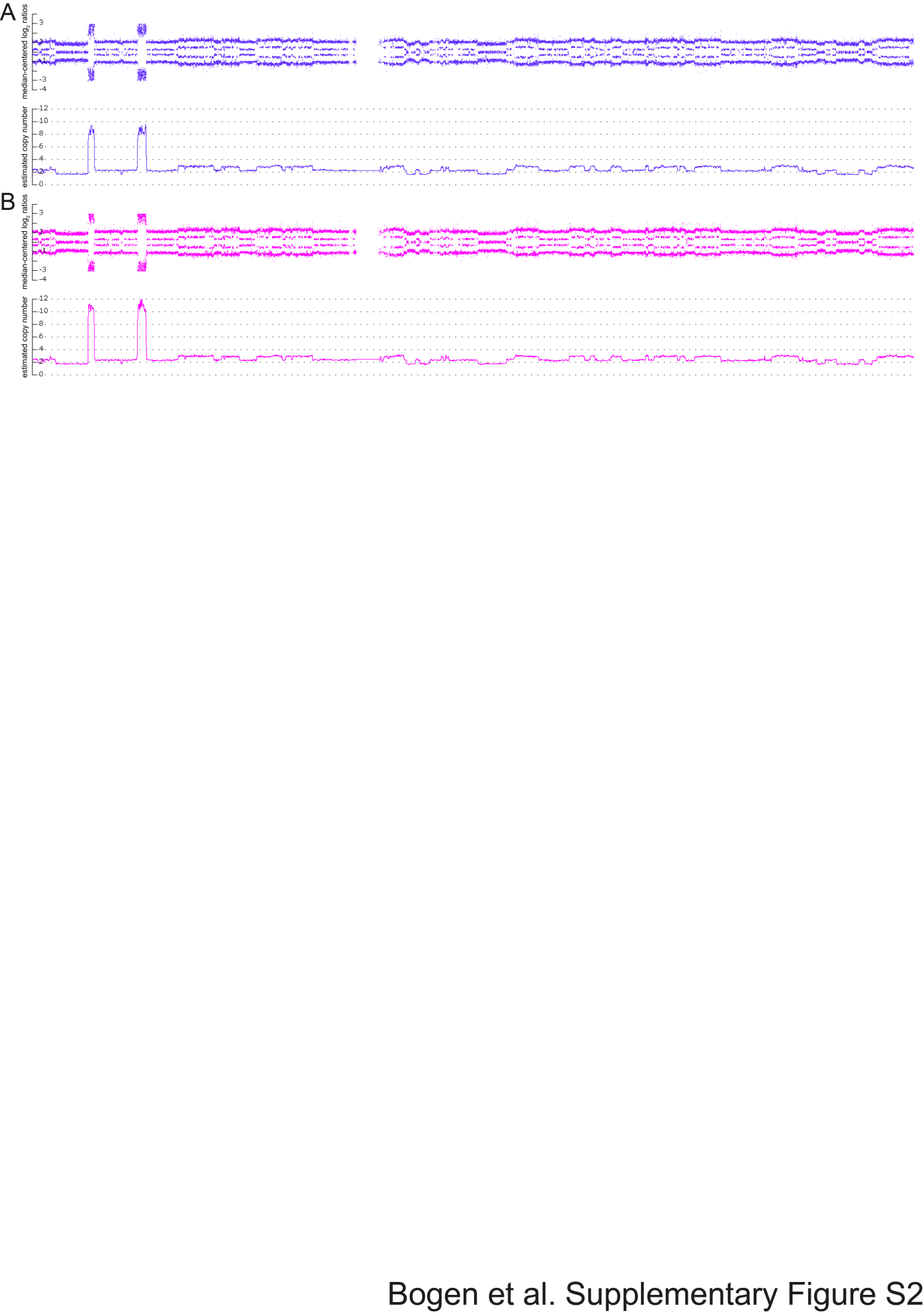


Matching chromothripsis profiles of chromosome 2 with *MYCN* and *ALK* amplification in tumors 1073-Tu a (a) and 1073-Tu b (b) of patient #5.

### Supporting Information Figure 4


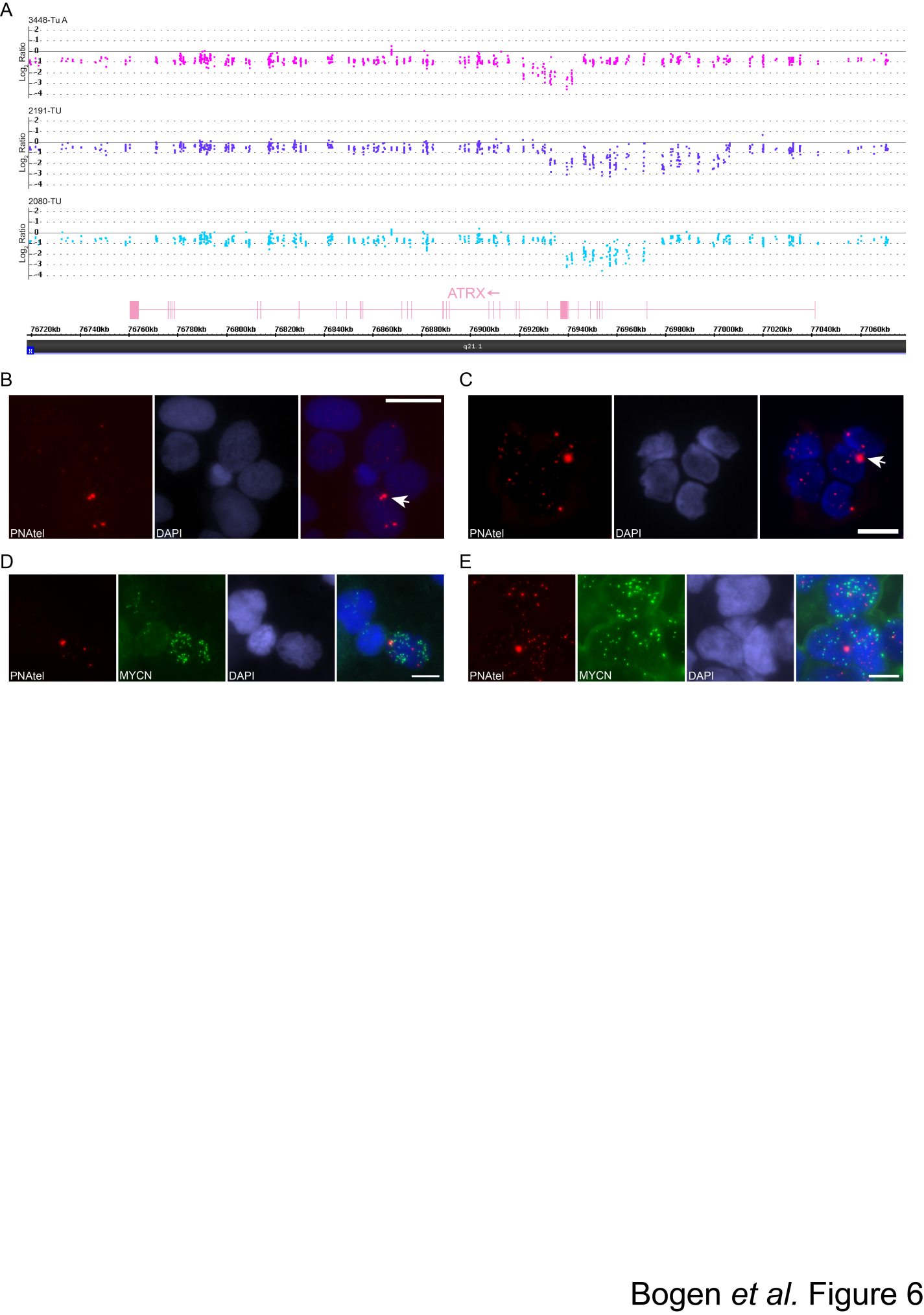


ATRX deletions in three hetMNA tumors of patients in the older cohort. SNP copy number profiles were generated using the ChAS software. The baseline is set to the average chromosomal copy number for a sample.

### Supporting Information Figure 5


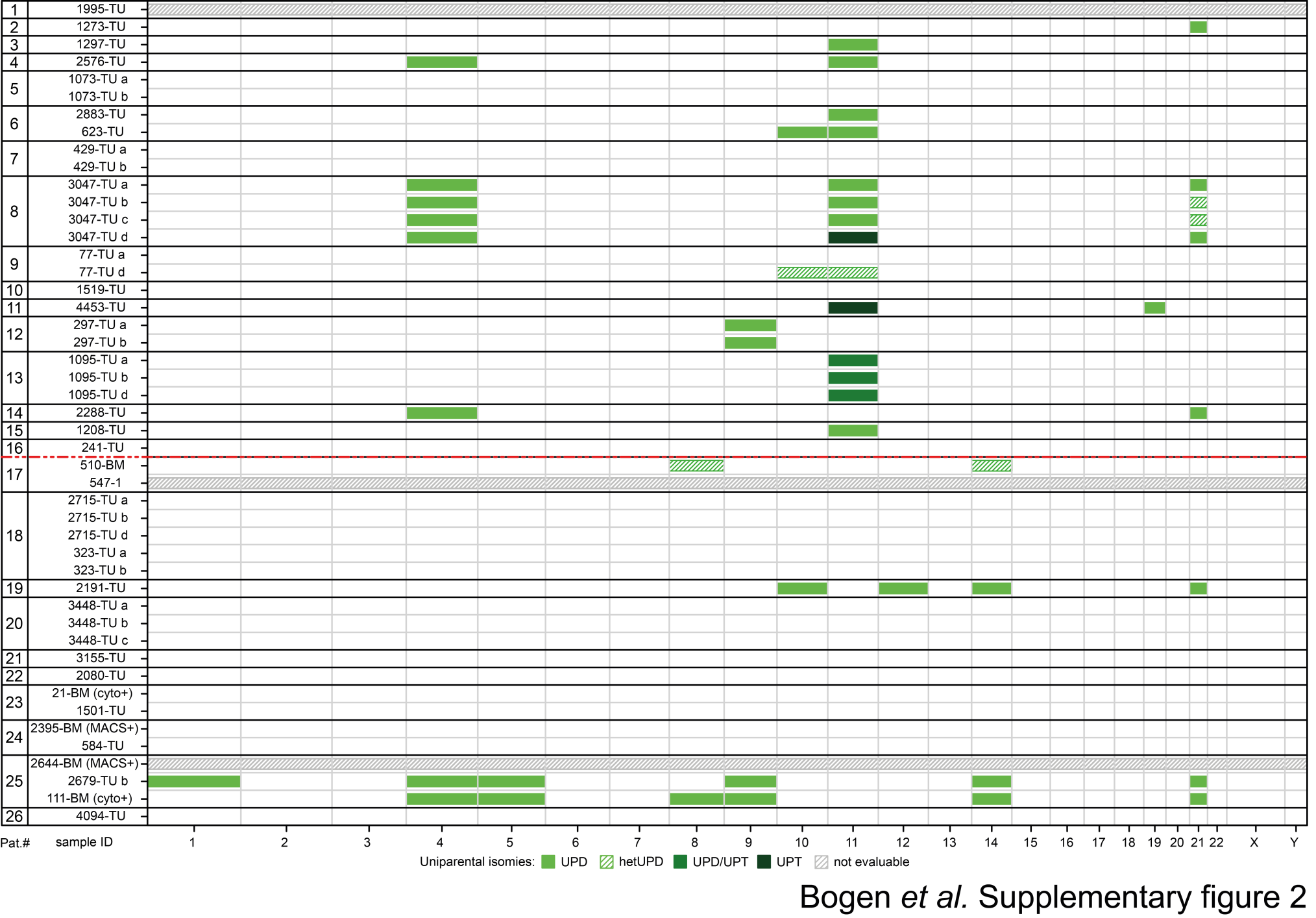


Summary of the uniparental whole chromosome aberrations detected by SNP array analysis in hetMNA NB samples by ascending patient age at diagnosis. The red dashed line marks the 18-month-age cutoff. Coloring of the segments as described in the figure legend is based on the copy number state of the chromosomes as determined by visual inspection of the SNP array data using the ChAS software. Grey striation of a sample indicated that the SNP array profile was not evaluable due to very low tumor cell content. BM, bone marrow sample; cyto+, DNA was extracted from BM cytospins; MACS+, DNA from GD2-positive cell fraction extracted by MACS from BM samples; TU, tumor sample; UPD, uniparental disomy; UPT, uniparental trisomy; hetUPD, heterogeneous UPD (UPD was only detected in a fraction of tumor cells in the respective sample).

### Supporting Information Figure 6


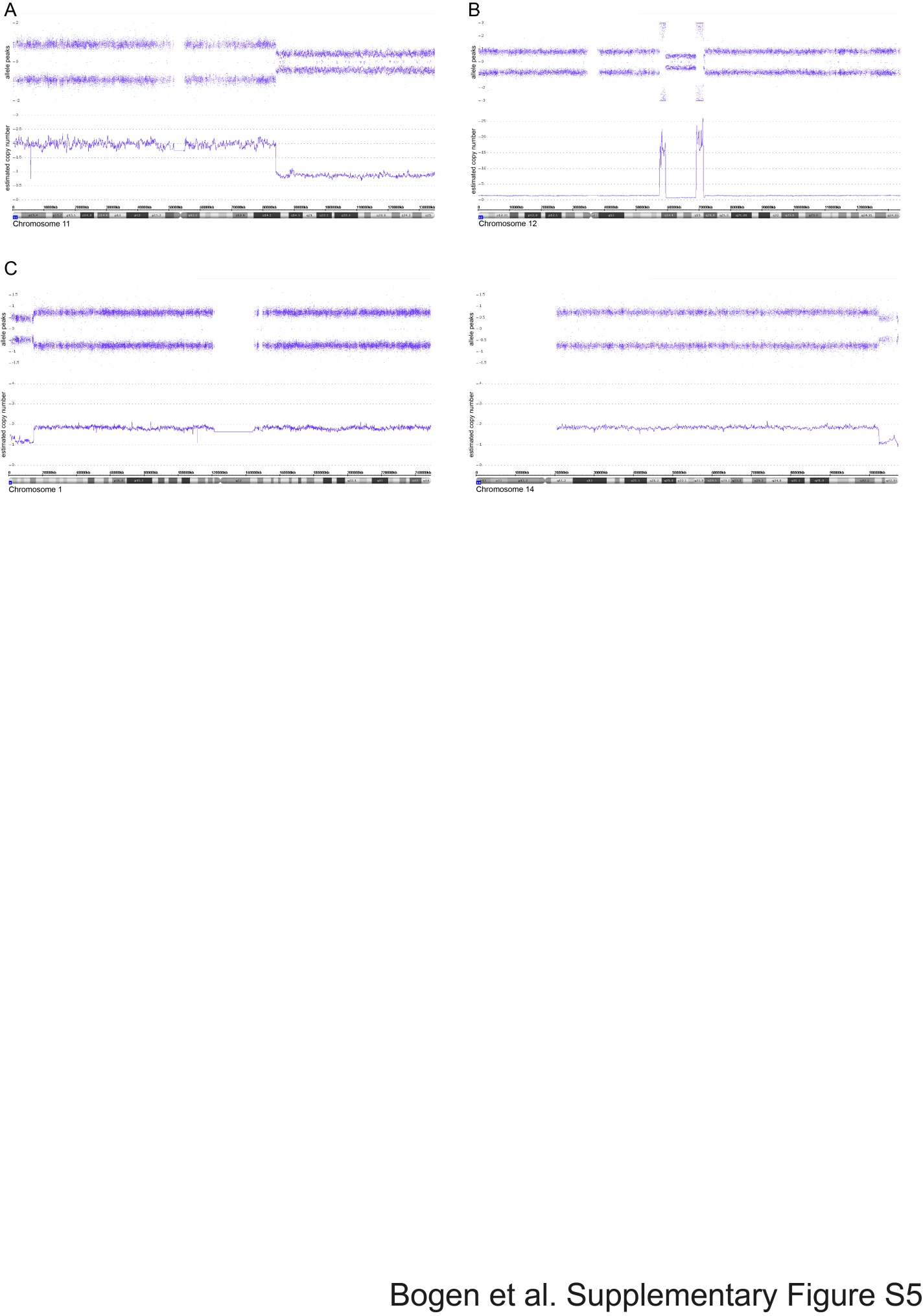


Examples of whole chromosome UPDs with segmental SCAs discovered in three patients by SNP genotyping: 3047-TU d (a), 2191-TU (b), and 2679-TU (c).

### Supporting Information Table 1

| **Patient ID** | **Sample ID** | **Type of sample** | **DNA index/ ploidy** | **tcc [%]** |
| --- | --- | --- | --- | --- |
| 1 | 1995-TU | TU post therapy | ne | 5 |
| 2 | 1273-TU | Primary TU at DX | near-triploid | >70 |
| 3 | 1297-TU | Primary TU at DX | near-triploid | 85 |
|  | 1297-PB | Const. ref. | diploid |  |
| 4 | 2576-TU | Primary TU | 1,41 | 80 |
| 5 | 1073-TU a | Primary TU at DX | 1,34 | 90 |
|  | 1073-TU b | Primary TU at DX | 1,34 | 90 |
| 6 | 2871-BM | Const. ref. | diploid |  |
|  | 2883-TU | Primary TU at DX | 1,49 | 75 |
|  | 623-TU | Second look | near-triploid | 60 |
| 7 | 370-BM | Const. ref. | diploid |  |
|  | 429-TU a | Primary TU at DX | 1,55 | 80-90 |
|  | 429-TU b | Primary TU at DX | 1,55 | 80-90 |
| 8 | 3047-TU a | Primary TU at DX | near-triploid | 70-90 |
|  | 3047-TU b | Primary TU at DX | near-triploid | 70-90 |
|  | 3047-TU c | Primary TU at DX | near-triploid | 70-90 |
|  | 3047-TU d | Primary TU at DX | near-triploid | 70-90 |
|  | 3343-PB | Const. ref. | diploid |  |
| 9 | 77-TU a | Primary TU at DX | near-triploid | 85-95 |
|  | 77-TU d | Primary TU at DX | near-triploid | 85-95 |
|  | 322-BM | Const. ref. | diploid |  |
| 10 | 1519-TU | Primary TU at DX | 1,64 | 70-90 |
| 11 | 4453-TU | BM at DX | aneuploid | >90 |
|  | 309-BM | Const. ref. | diploid |  |
| 12 | 297-TU a | Primary TU at DX | 1,36 | <40 |
|  | 297-TU b | Primary TU at DX | near-triploid | <20 |
|  | 58-BM | Const. ref. | diploid |  |
| 13 | 1095-TU a | Primary TU at DX | near-triploid | 80 |
|  | 1095-TU b | Primary TU at DX | near-triploid | 80 |
|  | 1095-TU d | Primary TU at DX | near-triploid | 80 |
|  | 1095-BM | Const. ref. | diploid |  |
| 14 | 2288-TU | Primary TU at DX | near-triploid | >90 |
|  | 2100-BM | Const. ref. | diploid |  |
| 15 | 1208-TU | Primary TU at DX | 1,46 | 80-90 |
| 16 | 241-TU | Primary TU at DX | aneuploid | 50-60 |
| 17 | 510-BM | BM at DX | aneuploid | >80 |
|  | 547-1 | Cell-free blood DNA at DX | aneuploid | 0 (DNA) |
| 18 | 2715-TU a | Primary TU at DX | diploid | 30 |
|  | 2715-TU b | Primary TU at DX | diploid | 40 |
|  | 2715-TU d | Primary TU at DX | diploid | 50 |
|  | 323-TU a | Lung metastasis | diploid | 30 |
|  | 323-TU b | Lung metastasis | diploid | 20 |
|  | 2780-PB | Const. ref. | diploid |  |
| 19 | 2191-TU | Primary TU at DX | near-triploid | 90 |
| 20 | 3448-TU a | Primary TU at DX | 1.0 (with polyploidization) | 80-95 |
|  | 3448-TU b | Primary TU at DX | 1.0 (with polyploidization) | 80-95 |
|  | 3448-TU c | Primary TU at DX | 1.0 (with polyploidization) | 80-95 |
|  | 1724-BM | Const. ref. | diploid |  |
| 21 | 3155-TU | Primary TU at DX | aneuploid (because of gains, baseline diploid) | 90 |
| 22 | 890-BM* | BM at DX | diploid | 22 tumor cells in 5,12x10^6 MNCs |
|  | 2080-TU | TU post therapy | aneuploid (because of gains, baseline diploid) | >80 |
| 23 | 21-BM (cyto+) | BM at DX | aneuploid | >30 |
|  | 1501-TU | TU post therapy | 2,01 | <50 |
|  | 1324-Pb | Const. ref. | diploid |  |
| 24 | 2395-BM (MACS+) | Enriched BM at DX | diploid (with polyploidization) | 20 |
|  | 584-TU | TU post therapy | diploid (with polyploidization) | <50 |
|  | 2395-BM | Const. ref. | diploid |  |
| 25 | 2644-BM (MACS+) | Enriched BM at DX | ? | 20-30 |
|  | 2679-TU b | Primary TU at DX | aneuploid | 95 |
|  | 111-BM (cyto+) | BM at relapse | aneuploid | >50 |
|  | 3148-Pb | Const. ref. | diploid |  |
| 26 | 4094-TU | Primary TU at DX | 1,10 | 90 |
|  | 0783-BM | Const. ref. | diploid |  |

Ploidy and tumor cell content (tcc) of samples examined in this study. Although patient #1 displayed signs of MNA in the SNP profile, tcc was too low to make any assumptions on DNA ploidy. *MNA detected by FISH but the sample was not displayed in the SNP array analysis due to low tcc and thus a flat profile. Abbreviations: TU, tumor; BM, bone marrow; Constitutional reference, Const. ref.; DX, diagnosis.

### Supporting Information Table 2

Segmental aberrations and amplicons discovered in the samples of the study cohort.

### Supporting Information Table 3

| **UPD/UPT occurrence** | | | | | | | | | |
| --- | --- | --- | --- | --- | --- | --- | --- | --- | --- |
| **aneuploid** | | | | | **di-/tetraploid** | | | | |
|  | | **UPD** | **no UPD** | **Total** |  |  | **UPD** | **no UPD** | **Total** |
| **non-MNA** | Count | 41 | 46 | 87 | **non-MNA** | Count | 1 | 70 | 71 |
|  | Expected Count | 45.0 | 42.0 | 87 |  | Expected Count | 0.6 | 70.4 | 71 |
|  | % within UPD occurrence | 47.1 | 52.9 | 100 |  | % within UPD occurrence | 1.4 | 98.6 | 100 |
| **hetMNA** | Count | 14 | 5 | 19 | **hetMNA** | Count | 0 | 5 | 5 |
|  | Expected Count | 9.8 | 9.2 | 19 |  | Expected Count | 0.0 | 5.0 | 5 |
|  | % within UPD occurrence | 73.7 | 26.3 | 100 |  | % within UPD occurrence | 0 | 100 | 100 |
| **homMNA** | Count | 7 | 7 | 14 | **homMNA** | Count | 0 | 31 | 31 |
|  | Expected Count | 7.2 | 6.8 | 14 |  | Expected Count | 0.3 | 30.7 | 31 |
|  | % within UPD occurrence | 50 | 50 | 100 |  | % within UPD occurrence | 0 | 100 | 100 |
| **Total** | Count | 62 | 58 | 120 |  | Count | 1 | 106 | 107 |
|  | Expected Count | 62.0 | 58.0 | 120 | **Total** | Expected Count | 1.0 | 106.0 | 120 |
|  | % within UPD occurrence | 51.7 | 48.3 | 100 |  | % within UPD occurrence | 0.9 | 99.1 | 100 |

Distribution of uniparental di- or trisomies in the patient cohort. Only samples with an evaluable SNP array profile and sufficient tumor cell content were included in the analysis.
